# Chronic Disease in Health Emergencies: In the Eye of the Hurricane

**Published:** 2006-03-15

**Authors:** Earl S Ford, Ali H Mokdad, Michael W Link, William S Garvin, Lisa C McGuire, Jiles Ruth B, Lina S Balluz

**Affiliations:** Division of Adult and Community Health, National Center for Chronic Disease Prevention and Health Promotion, Centers for Disease Control and Prevention; Division of Adult and Community Health, National Center for Chronic Disease Prevention and Health Promotion, Centers for Disease Control and Prevention, Atlanta, Ga; Division of Adult and Community Health, National Center for Chronic Disease Prevention and Health Promotion, Centers for Disease Control and Prevention, Atlanta, Ga; Division of Adult and Community Health, National Center for Chronic Disease Prevention and Health Promotion, Centers for Disease Control and Prevention, Atlanta, Ga; Division of Adult and Community Health, National Center for Chronic Disease Prevention and Health Promotion, Centers for Disease Control and Prevention, Atlanta, Ga; Division of Adult and Community Health, National Center for Chronic Disease Prevention and Health Promotion, Centers for Disease Control and Prevention, Atlanta, Ga; Division of Adult and Community Health, National Center for Chronic Disease Prevention and Health Promotion, Centers for Disease Control and Prevention, Atlanta, Ga

## Abstract

**Introduction:**

Inadequately controlled chronic diseases may present a threat to life and well-being during the emergency response to natural disasters. An estimate of the possible numbers of people who may require treatment for chronic diseases should help in planning a response, but such information for local areas is not easily accessible. We explored how a current surveillance system could be used to provide estimates of the potential needs for emergency treatment of chronic diseases in the wake of a natural disaster.

**Methods:**

We used data from adults aged 18 years or older who participated in the Behavioral Risk Factor Surveillance System (BRFSS) in 2004 to estimate the prevalence and numbers of people with diabetes, heart disease, stroke, hypertension, and current asthma who lived in the New Orleans–Metairie–Kenner, La, metropolitan statistical area.

**Results:**

About 9.0% of participants had diabetes, 4.6% had angina or coronary heart disease, 3.0% had had a myocardial infarction, 2.0% had had a stroke, and 6.3% had current asthma. About 25.4% adults had at least one of the above conditions.

**Conclusion:**

A surveillance system such as the BRFSS can provide potentially useful baseline information about the numbers of people with chronic diseases and the treatment that they receive; this information can assist the medical and public health community in assessing the needs of people with chronic diseases after disasters and in planning relief efforts.

## Introduction

After Hurricane Katrina roared through the Gulf Coast region on August 29, 2005, the immediate concerns among the public health and medical community were infectious diseases, injuries, and environmental risks, and the overriding goal was preservation of life. However, with the devastation of the regional infrastructure, including the medical sector, and the subsequent large-scale displacement of residents from the affected areas, treatment of chronic diseases emerged as a critical concern. Inadequately controlled chronic diseases may present a threat to life and well-being in the immediate wake of natural disasters, but their treatment traditionally has not been recognized as a public health or medical priority. In interviews with medical personnel in hurricane-affected areas, a leading concern expressed was the urgency of treating people with chronic diseases such as diabetes, cardiovascular disease, hypertension, and kidney disease.

Publications on natural disasters provide little information about treating large numbers of people with chronic diseases; furthermore, these texts primarily reflect experiences of dealing with natural disasters in poor countries (1-5). In wealthier countries with aging populations, the need to address treatment of chronic diseases after a natural disaster requires reevaluation, especially in situations where the medical infrastructure is severely disrupted.

An estimate of the possible numbers of residents who may require treatment for chronic diseases should help to inform the emergency response to natural disasters. Furthermore, information about types of treatment received by patients with chronic diseases and medical equipment used by them may allow emergency response planners to stock and ship appropriate quantities of medical supplies. However, such information for local areas is not easily accessible. Our objective was to explore how a current surveillance system could be used to estimate the potential needs for emergency treatment of chronic diseases in the wake of a natural disaster.

## Methods

Data for this analysis are from the Behavioral Risk Factor Surveillance System (BRFSS) conducted in 2004 (6). State health agencies selected an independent probability sample from noninstitutionalized adults in the United States aged 18 years or older with telephones by using a multistage sampling design. All states used an identical core questionnaire administered over the telephone by trained interviewers. In addition, states administered additional optional modules. The survey was approved by an institutional review board in each participating state.

Using definitions of metropolitan/micropolitan statistical areas (MMSAs) produced by the U.S. Census Bureau, a new data set was created that included MMSAs with at least 500 respondents (7). To produce estimates for these geographic areas, the final state-level weight was re-poststratified to reflect the adult population for each MMSA. This analysis is limited to the MMSA of New Orleans–Metairie–Kenner, La, which includes Jefferson Parish, Orleans Parish, St. Tammany Parish, Plaquemines Parish, St. Bernard Parish, St. Charles Parish, and St. John the Baptist Parish.

The BRFSS survey used in this analysis asked participants about a selected number of chronic conditions. The questionnaire included information about the following chronic diseases: diabetes, cardiovascular disease (myocardial infarction, angina or coronary heart disease, and stroke), hypertension, and asthma. Participants were asked, "Have you ever been told by a doctor that you have diabetes?" If the answer was yes and the participant was female, she was asked, "Was this only when you were pregnant?" Some respondents said they were told they had prediabetes or borderline diabetes. Possible responses included 1) yes; 2) yes, but female told only during pregnancy; 3) no; and 4) no, prediabetes or borderline diabetes. Participants who answered yes were considered to have diagnosed diabetes. Participants who provided one of the other three answers were not considered to have diagnosed diabetes. In addition, participants with diabetes were asked whether they used oral hypoglycemic medications or insulin.

Respondents were also asked the following question: "Has a doctor, nurse, or other health professional ever told you that you had any of the following: 1) a heart attack, also called a myocardial infarction, 2) angina or coronary heart disease, or 3) a stroke?" Participants who answered yes were considered to have the condition. Participants who answered yes to the question, "Have you ever been told by a doctor, nurse, or other health professional that you have high blood pressure?" were considered to have hypertension. In addition, these respondents were asked whether they were currently taking medication for their hypertension.

Respondents who responded yes to the questions, "Did a doctor ever tell you that you had asthma?" and "Do you still have asthma?" were classified as having current asthma. Respondents who answered yes to the first question but no to the second one were classified as having former asthma. Respondents who reported not having been told by a physician that they had asthma were classified as never having had asthma.

We used SUDAAN version 8.0 (Research Triangle Institute, Research Triangle Park, NC) to account for the complex sampling design of the survey. We present estimates of the percentages (with 95% confidence intervals [CIs]) and the estimated numbers of participants with chronic conditions stratified by age, sex, race and ethnicity, and education level. For participants who reported having a chronic condition, we also present estimates of the percentages (with CIs) and the estimated numbers who reported receiving treatment for their condition.

## Results

A total of 1681 respondents representing 977,294 adults aged 18 years or older from the New Orleans–Metairie–Kenner MMSA were available for 2004. Participants ranged in age from 18 to 92 years, 36% were men, 64% were white, 28% were African American, 4% were Hispanic, 4% were other races and ethnicities, and 10% had not completed high school.

About 9.0% (representing an estimated 87,944 people) of the adult population within the New Orleans–Metairie–Kenner MMSA reported that they had diabetes (Table 1). Among respondents with diagnosed diabetes, 79.4% (or an estimated 69,792 adults) reported using oral glucose-lowering medications, and 24.6% (or an estimated 21,643 adults) reported using insulin (Table 2).

About 4.6% (representing an estimated 42,271 adults) reported that they had angina or coronary heart disease, 3.0% that they had had a myocardial infarction (an estimated 28,030 adults), and 2.0% that they had had a stroke (an estimated 18,178 adults). In addition, 29.0% reported having hypertension (an estimated 270,176 adults). Of those, 81.1% (an estimated 219,050) reported using antihypertensive medications. No information about the use of medications other than aspirin to treat cardiovascular disease was collected in this survey.

About 12% (or an estimated 119,375 adults) reported ever having asthma and 6.3% (or an estimated 61,645 adults) had current asthma. Among adults with current asthma, 57.7% (or an estimated 30,776 adults) reported using medications during the previous 30 days to treat their asthma. In addition, of adults with current asthma, 38.9% (or an estimated 22,578 adults) were not taking asthma medications, 15.5% (or an estimated 8990 adults) took medications less than once per week, 5.0% (or an estimated 2880 adults) took medications once or twice per week, 1.9% (or an estimated 1095 adults) took medications more than twice per week but not every day, 15.4% (or an estimated 8947 adults) took medications once every day, 15.3% (or an estimated 8865 adults) twice or more every day, and 8.2% (or an estimated 4763 adults) were not sure or did not know.

About 25.4% (95% CI, 22.8%–28.2%) or an estimated 233,876 adults had at least one of the mentioned conditions: 15.6% (an estimated 143,545 adults) had one condition, 8.4% (an estimated 77,396 adults) had two conditions, 1.1% (an estimated 10,371 adults) had three conditions, and 0.3% (an estimated 2563 adults) had four or more conditions.

## Discussion

Hurricane Katrina made it clear that the treatment of chronic diseases after a natural disaster should be a public health and medical priority. Immediately after a disaster, rescue efforts are critically important. However, in the following days, the unmet needs of patients with chronic diseases may become a threat to their lives and well-being. The need to treat chronic conditions is especially magnified when there are catastrophic disruptions of the medical infrastructure, including pharmacies, when access to medical care and medications is severely compromised or totally cut off, and when large-scale evacuations of the population occur.

Previous research has indicated that natural disasters may be followed by increases in myocardial infarction (8,9). In addition, changes in behaviors that affect the risk for chronic diseases may occur in the wake of disasters. According to one study, respondents in Connecticut, New Jersey, and New York were more likely to drink more alcohol and smoke more as a result of the September 11 attacks on the World Trade Center (10).

Little has been published about treating chronically ill people during disasters. Perhaps this is because many of the disasters have occurred in poor countries where chronic disease has been historically less of a health priority. Or perhaps in wealthier countries, catastrophic damage to the medical infrastructure is uncommon, so patients with chronic diseases continue to receive care. One investigation of St. Thomas, Virgin Islands, after it was devastated by Hurricane Marilyn, found that antihypertensive medications and insulin-loaded syringes topped the list of needs among the elderly (11). In addition, a special operations response team treating people affected by Hurricane Andrew in Florida rapidly exhausted its supplies of insulin (12). The initial impressions from news reports on Hurricane Katrina seemed consistent with these findings. Research indicates that adverse effects on glycemic control and quality of life among people with diabetes (13,14), risk of hypertension, coronary heart disease (9), and other chronic conditions are possible after a natural disaster.

Ensuring access to adequate medical supplies is integral to successful disaster relief efforts. For example, as part of the response to Hurricane Katrina, many doses of insulin were rushed to the Louisiana and Mississippi coastal areas. In the future, medications for treating chronic diseases may also need to be moved quickly to affected areas.

The results from our analysis suggest that a surveillance system such as the BRFSS may provide data that are critical to planning for natural or man-made disasters. Previously, the flexible design of the BRFSS allowed states to add questions to their ongoing surveys to address changing situations and crises, such as the World Trade Center attacks (10). Enhancements to the surveillance system would increase its value. For example, in-depth questionnaires about additional chronic conditions such as chronic pulmonary diseases (15), treatments being used, supplies of medicines typically on hand, and access to health services or treatment facilities would allow more detailed profiles to be developed. In addition, larger sample sizes would produce better, more stable, and more detailed estimates. Estimates would need to be updated periodically because of changes in population size, changes in the prevalence of chronic conditions, and changes in medical treatment patterns. In addition, surveillance efforts could provide valuable data about changes in the prevalence of chronic conditions or their risk factors in the intermediate and long term.

Limitations of our study include the inability to restrict analyses to specific geographic areas. In addition, sample sizes were too small to allow multiple stratifications and produce robust estimates. Both of these limitations could be addressed by altering sampling frames and increasing sample sizes. Only a limited set of questions about chronic conditions was included in the BRFSS. All information was based on self-reports. However, studies have shown good reliability and validity for questions related to chronic conditions (16). Although telephone coverage is high in the United States, in selected areas, including those with high poverty rates, telephone coverage may be below average. Finally, institutionalized people, including people in hospitals or nursing homes, were not interviewed.

In areas that are at risk for future natural disasters, having baseline information about the numbers of people with chronic diseases and the treatment they receive should assist the medical and public health community in assessing the needs of people with chronic diseases after such disasters and in planning future relief efforts. The findings in this report document the widespread needs among people with chronic diseases in the New Orleans–Metairie–Kenner MMSA that was ravaged by a hurricane and flooding. Our findings show that there is a need to monitor the short- and long-term physical health of the affected population. Establishing a comprehensive understanding of the medical and chronic disease needs of communities during emergencies should arm public health professionals with the critical information needed to prepare for medical care of people with chronic diseases after a disaster.

## Figures and Tables

**Table 1:** Unadjusted Prevalence and Estimated Numbers of Selected Chronic Conditions Among Adults Aged 18 or Older Within the New Orleans–Metairie–Kenner, La, Metropolitan/Micropolitan Statistical Area, Behavioral Risk Factor Surveillance System, 2004

	**Diabetes**		**Angina or Coronary Heart Disease**		**Myocardial Infarction**		**Stroke**		**Hypertension**		**Current Asthma**	

	**% (95% CI^a^)**	**Estimated No.**	**% (95% CI^a^)**	**Estimated No.**	**% (95% CI^a^)**	**Estimated No.**	**% (95% CI^a^)**	**Estimated No.**	**% (95% CI^a^)**	**Estimated No.**	**% (95% CI^a^)**	**Estimated No.**

**Total**	9.0 (7.6-10.7)	87,944	4.6 (3.5-5.9)	42,271	3.0 (2.2-4.1)	28,030	2.0 (1.4-2.8)	18,178	29.0 (26.4-31.7)	270,176	6.3 (5.0-7.9)	61,645

**Age, y**												

18-44	2.1 (1.3-3.6)	10,812	—b	—	—b	—	—b	—	11.4 (9.0-14.4)	54,609	6.7 (4.8-9.3)	33,592
45-64	14.7 (11.6-18.4)	46,641	6.7 (4.7-9.6)	20,154	3.9 (2.4-6.3)	11,698	3.0 (1.8-5.1)	9,109	40.4 (35.6-45.4)	121,527	5.4 (3.8-7.6)	17,248
≥65	19.9 (15.0-25.8)	29,675	9.3 (6.2-13.6)	13,536	9.7 (6.5-14.3)	14,269	5.2 (2.9-9.1)	7,599	62.2 (55.4-68.6)	91,744	—b	—

**Sex**												

Male	8.1 (5.9-11.0)	36,935	5.9 (4.0-8.6)	25,339	4.3 (2.9-6.3)	18,485	—b	—	29.8 (25.7-34.3)	128,762	5.2 (3.4-7.8)	23,600
Female	9.8 (8.0-12.0)	51,009	3.4 (2.4-4.8)	16,932	1.9 (1.2-3.0)	9,545	2.1 (1.4-3.4)	10,650	28.3 (25.2-31.6)	141,413	7.3 (5.6-9.5)	38,045

**Race or ethnicity**												

White	8.0 (6.3-10.2)	43,511	5.5 (4.0-7.5)	28,738	3.7 (2.6-5.3)	19,304	2.6 (1.7-4.0)	13,650	28.6 (25.4-31.9)	149,777	5.0 (3.6-6.9)	27,203
African American	11.0 (8.3-14.4)	35,579	3.6 (2.0-6.3)	10,919	—b	—	—b	—	32.6 (27.7-38.0)	99,857	8.5 (5.9-12.1)	27619
Hispanic	—b	—	—b	—	—b	—	—b	—	—b	—	—b	—
Other	—b	—	—b	—	—b	—	—b	—	25.3 (14.3-40.7)	11,425	—b	—

**Education**												

Less than high school	14.9 (9.9-21.8)	15,101	—b	—	9.6 (5.5-16.3)	8,973	—b	—	47.8 (38.8-57.0)	45,046	9.3 (5.4-15.6)	9,503
High school graduate	8.3 (6.8-10.0)	72,071	4.4 (3.3-5.9)	36,864	2.3 (1.6-3.2)	19,057	2.0 (1.3-3.0)	16,640	26.8 (24.2-29.6)	224,147	5.9 (4.6-7.6)	51,461

a CI indicates confidence interval.

b Estimates do not meet criteria for reliability or precision.

**Table 2:** Unadjusted Prevalence and Estimated Numbers of Participants by Treatment Received Among Adults Aged 18 or Older With Selected Chronic Conditions Within the New Orleans–Metairie–Kenner, La, Metropolitan/Micropolitan Statistical Area, Behavioral Risk Factor Surveillance System, 2004

**Condition**	**Treatment**	**% (95% CI^a^)**	**Estimated No.**
Diabetes	Oral glucose-lowering medications	79.4 (71.1-85.8)	69,792
	Insulin	24.6 (18.0-32.6)	21,643
Angina or coronary heart disease	Aspirin	72.6 (58.5-83.3)	29,136
Myocardial infarction	Aspirin	89.1 (77.3-95.2)	24,232
Stroke	Aspirin	67.9 (49.2-82.1)	12,336
Hypertension	Antihypertensive medications	81.1 (76.5-85.0)	219,050
Current asthma	Asthma medication during past 30 days	57.7 (44.3-70.0)	30,776

a CI indicates confidence interval.
